# Neuroradiological Evaluation of Anatomo-Morphometric Arcuate Fascicle Modifications According to Different Brain Tumor Histotypes: An Italian Multicentric Study

**DOI:** 10.3390/brainsci15060625

**Published:** 2025-06-10

**Authors:** Roberto Altieri, Andrea Bianconi, Stefano Caneva, Giovanni Cirillo, Fabio Cofano, Sergio Corvino, Oreste de Divitiis, Giuseppe Maria Della Pepa, Ciro De Luca, Pietro Fiaschi, Gianluca Galieri, Diego Garbossa, Giuseppe La Rocca, Salvatore Marino, Edoardo Mazzucchi, Grazia Menna, Antonio Mezzogiorno, Alberto Morello, Alessandro Olivi, Michele Papa, Daniela Pacella, Rosellina Russo, Giovanni Sabatino, Giovanna Sepe, Assunta Virtuoso, Giovanni Vitale, Rocco Vitale, Gianluigi Zona, Manlio Barbarisi

**Affiliations:** 1 Multidisciplinary Department of Medical-Surgical and Dental Specialties, University of Campania “Luigi Vanvitelli”, 80131 Naples, Italy; roberto.altieri.87@gmail.com (R.A.); manlio.barbarisi@unicampania.it (M.B.); 2 Department of Neuroscience, Rehabilitation, Ophthalmology, Genetics, Maternal and Child Health (DINOGMI), University of Genova, 16132 Genova, Italy; andrea.bianconi@unige.it (A.B.); canevastefano@gmail.com (S.C.); pietro.fiaschi@unige.it (P.F.); gianluigi.zona@hsanmartino.it (G.Z.); 3 Department of Neurosurgery, IRCCS Ospedale Policlinico San Martino, 16132 Genova, Italy; 4 Laboratory of Morphology of Neuronal Network, Department of Public Medicine, University of Campania “Luigi Vanvitelli”, 80138 Naples, Italy; giovanni.cirillo@unicampania.it (G.C.); ciro.deluca@unicampania.it (C.D.L.); antonio.mezzogiorno@unicampania.it (A.M.); michele.papa@unicampania.it (M.P.); giovanna.sepe@unicampania.it (G.S.); assunta.virtuoso@unicampania.it (A.V.); 5 Neurosurgery Unit, Department of Neuroscience “Rita Levi Montalcini”, University of Turin, Via Cherasco, 15, 10126 Turin, Italy; fabio.cofano@unito.it (F.C.); diego.garbossa@unito.it (D.G.); alberto.morello@unito.it (A.M.); 6 Department of Neurosurgery, “Ospedale del Mare” Hospital, 80147 Naples, Italy; 7 Department of Neuroscience and Reproductive and Odontostomatological Sciences, Neurosurgical Clinic, School of Medicine, University of Naples “Federico II”, Via Pansini, 5, 80131 Naples, Italy; oreste.dedivitiis@unina.it; 8 Institute of Neurosurgery, Fondazione Policlinico Universitario A. Gemelli IRCCS, Catholic University, 00168 Rome, Italy; giuseppemaria.dellapepa@policlinicogemelli.it (G.M.D.P.); gianluca.galieri@gmail.com (G.G.); giularocca86@gmail.com (G.L.R.); salvo.marino130592@gmail.com (S.M.); mennagrazia@gmail.com (G.M.); alessandro.olivi@policlinicogemelli.it (A.O.); giovanni.sabatino@policlinicogemelli.it (G.S.); 9 Neurosurgical Training Center and Brain Research, Mater Olbia Hospital, 07026 Olbia, Italy; edoardo.mazzucchi@materolbia.com; 10 Department of Neurosurgery, IRCCS Regina Elena National Cancer Institute, 00144 Rome, Italy; 11 Department of Public Health, University of Naples “Federico II”, 80138 Naples, Italy; daniela.pacella@unina.it; 12 Department of Radiology, Neuroradiology Unit, Fondazione Policlinico Universitario A. Gemelli IRCCS, 00136 Rome, Italy; rosellina.russo@policlinicogemelli.it; 13 Neurosurgery Unit, Regional Hospital San Carlo, 85100 Potenza, Italy; gianninovitale@alice.it; 14 Division of Neurosurgery, “Ospedale del Mare” Hospital, 80147 Naples, Italy; rocky84@hotmail.it

**Keywords:** glioblastoma, low-grade glioma, metastasis, meningioma, arcuate fascicle, connectome

## Abstract

**Background:** The arcuate fasciculus (AF) is a critical white matter (WM) tract that connects key cortical language-processing regions, including the so-called Broca’s and Wernicke’s areas. The aim of the present study was to quantitatively assess its radiological–anatomical–morphometric modifications according to different brain tumor histotypes. **Methods:** A retrospective multicentric Italian study was conducted. AF reconstructions were calculated for both hemispheres for each patient diagnosed with glioblastoma (GBM), low-grade glioma (LGG), brain metastasis, and meningioma using Elements Fibertracking 2.0 software (Brainlab AG, Munich, Germany). A 3D object of each fascicle was evaluated for its volume, average fractional anisotropy (FA), and length. The cerebral healthy hemisphere was compared to the pathological contralateral in different tumor histotypes. **Results:** In total, 1294 patients were evaluated. A total of 156 met the inclusion criteria. We found a significant difference between healthy hemisphere and the contralateral for AF mean length and volume (*p* = 0.01 and *p* < 0.001, respectively). Considering separately the different tumor histotypes, the GBM subgroup (98, 63%) confirmed the results for mean FA and volume (*p*-value < 0.001); LGG patients (26, 17%) showed no significant difference between healthy and pathological hemisphere for AF mean length, mean FA, and volume (*p*-value 0.5, *p*-value 0.3, *p*-value <0.1, respectively). In patients affected by brain metastasis (18, 12%), Student’s *t*-test showed a significant difference for FA (*p*-value 0.003). No differences were found in patients affected by meningiomas (14, 9%) (14). **Conclusions:** Thorough knowledge of the microscopic anatomy and function of the arcuate fasciculus, as well as the pattern of growth of the different brain tumor histotypes, along with a careful preoperative neuroradiological assessment are mandatory to plan a tailored surgical strategy and perform a safe and effective surgical technique. The AF could be displaced and infiltrated/destructed by the solid component and peritumoral edema, respectively, of GBM. LGG shows a prevalent infiltrative pattern. Metastases account for AF dislocation due to peritumoral edema. Meningiomas do not affect WM anatomy.

## 1. Introduction

**Figure 1:**
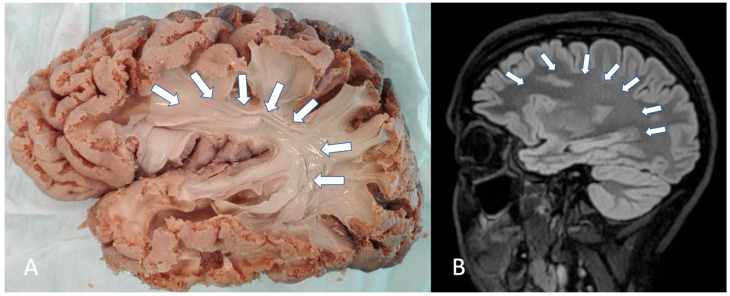
(**A**) Lateral perspective of the arcuate fasciculus (AF) in a human brain specimen (indicated by white arrows). The white matter dissection was conducted in an anatomical laboratory using an adult human brain preserved with the Klingler technique. Cortical layers were gently removed using a blunt spatula, followed by a stepwise fiber dissection proceeding from lateral to medial. (**B**) Sagittal T2-weighted magnetic resonance imaging (MRI) slice illustrating the AF (arrows).

The arcuate fasciculus (AF) is a critical white matter (WM) tract that connects key cortical language-processing regions, including the so-called Broca’s and Wernicke’s areas. Its integrity is fundamental for effective communication and cognitive function [1,2] (Figure 1). In patients with brain tumors, especially those located near perisylvian regions, the AF may undergo significant morpho-volumetric alterations, which can lead to language deficits and other cognitive impairments [3].

This study is grounded in the premise that different brain tumor histotypes exhibit distinct growth patterns, which in turn leads to variable alterations in the surrounding brain parenchyma. For example, glioblastomas (GBMs) typically originate within the WM and display a highly infiltrative yet rapid growth pattern, often resulting in both parenchymal invasion/disruption and mass effect, with the consequent compression and displacement of adjacent structures [4]. In contrast, lower-grade gliomas (LGGs) grow more slowly and infiltratively, allowing for greater neuroplastic adaptation and typically lacking a significant mass effect [5]. Metastases, despite recent evidence suggesting close interactions with the white matter and potential microinfiltrative behavior, are generally characterized by a macroscopically compressive growth pattern [6,7]. Meningiomas, on the other hand, exert pressure on the cortex from an extrinsic origin and grow slowly, typically causing displacement rather than infiltration [8].

Traditional neuroimaging methods have primarily focused on gross anatomical changes; however, advances in morpho-volumetric analysis now allow for a detailed quantitative assessment of WM tracts [9,10,11,12,13]. This approach provides deeper insights into how tumor-induced mass effects and infiltrative processes alter the integrity, volume, and shape of the AF. Moreover, different histotypes differ in terms of their growing pattern and interplay with nearby brain tissue [4,14,15,16,17,18,19]. In a previous paper, we demonstrated a specific alteration to Inferior Fronto-Occipital Fascicle (IFOF) across different brain tumors. Despite emerging research on functional reorganization in tumor-affected brains, the specific morpho-volumetric characteristics of the AF in this populations remain underexplored.

This study would like to address a significant gap in the current literature concerning morphometric alterations of the AF, building upon our previous investigations into other WM tracts, such as the IFOF. While many existing studies have focused on macroscopic anatomy through cadaveric dissection, functional mapping, or diffusion tensor imaging (DTI), few have explored the morphometric relationship between the AF and different histological tumor subtypes. In this study, we aim to quantify anatomo-morphometric changes—including tract volume, average fractional anisotropy (FA), and length—in the arcuate fasciculus of patients with various brain tumors. By comparing these parameters to those of healthy controls, we seek to elucidate the relationship between tumor pathology and WM tract integrity. We expect our findings to provide valuable insights to support pre-surgical planning and improve prognostic evaluations in neuro-oncology, ultimately contributing to more personalized and effective treatment strategies.

## 2. Materials and Methods

### 2.1. Study Design and Patient Population

This retrospective multicenter study was conducted across six Italian neurosurgical centers: two in the northern region (University of Turin; IRCCS Ospedale Policlinico San Martino, Genoa, Italy), two in the central region (Università Cattolica del Sacro Cuore, Rome; Mater Olbia Hospital, Olbia, Italy), and two in the southern region (University of Campania “Luigi Vanvitelli”, Naples; A.O.R. San Carlo, Potenza, Italy).

This study was conducted in accordance with the Declaration of Helsinki.

We reviewed the clinical records of 1294 patients who underwent surgery for intracranial tumors between January and December 2023. Inclusion criteria comprised (i) age ≥ 18 years; (ii) histopathological and/or molecular diagnosis of glioblastoma (GBM), low-grade glioma (LGG), brain metastases, or WHO grade I meningiomas; (iii) availability of preoperative contrast-enhanced MRI, including T1-weighted, FLAIR, and DTI sequences; and (iv) tumor proximity to the arcuate fasciculus (AF).

Patients were classified into four groups based on tumor histology: GBM (Group A), LGG (Group B), metastases (Group C), and meningiomas (Group D).

### 2.2. MRI Acquisition and Tractography Analysis

MRI scans were acquired using either 1.5 Tesla (A.O.R. San Carlo) or 3 Tesla systems (academic centers). DTI protocols varied: 32 directions (University of Turin, A.O.R. San Carlo, University of Campania, Mater Olbia Hospital), 60 directions (IRCCS San Martino), and 64 directions (Università Cattolica). Preoperative fiber tractography of the AF was performed using Elements Fibertracking and SmartBrush V. 2.0 (Brainlab AG) by neurosurgeons with proved neuro-oncological expertise. Regions of interest (ROIs) were manually drawn based on anatomical landmarks, as described by Feconja et al., targeting the subcallosal and ventral premotor cortices anteriorly, and the lateral occipital cortex posteriorly. Deterministic tractography was applied with a minimum streamline length of 50 mm and a fractional anisotropy (FA) threshold of 0.15. Only streamlines traversing the extreme capsule were retained, excluding fibers belonging to adjacent bundles. Fiber bundles were normalized to a standard anatomical template. The results were then reviewed by an expert interdisciplinary team of neuroradiologists and neuroanatomists. For each hemisphere, we measured fascicle volume, mean FA, and length. Comparisons were made between the pathological and healthy sides.

### 2.3. Statistical Analysis

Quantitative variables are reported as mean ± standard deviation, while categorical variables are presented as absolute and relative frequencies. Student’s paired *t*-tests were used to compare AF characteristics between hemispheres. Determinants of paired mean differences were analyzed using mixed-effects linear regression models, incorporating patient ID as a random effect in order to account for the effect and variability of each patient’s characteristics on the relationship between the determinants and the outcomes. For all models, significant confounders were identified using crude models and then added to the adjusted models. Statistical significance was set at *p* < 0.05. Analyses were performed using R software (v4.4.0).

## 3. Results

In the selected period, 1294 patients underwent neuro-oncological surgery at the abovementioned institutions. Among these patients, 156 met the inclusion criteria. In total, 98 (63%) were affected by GBM, 26 (17%) by low-grade glioma (LGG), 14 (9.0%) had meningioma, and 18 (12%) had brain metastasis.

In total, 83 patients were males (53%) and 73 were females (47%). A total of 109 (70%) patients had a tumor located on the left cerebral hemisphere, while the remaining 47 (30%) had a tumor on the contralateral side. All patients had a tumor harboring in proximity of the AF according to the inclusion criteria. The mean age was 59.

All demographic data are summarized in Table 1.

The mean volume of the enhancing nodule (EN) was 25 cm^3^, while the mean FLAIR volume beyond EN was 49 cm^3^.

The mean length of the AF of the healthy side and contralateral side was 102 mm and 100 mm, respectively. The mean FA of the healthy side and contralateral side was 0.46 and 0.44, respectively. The mean AF volume of the healthy side and contralateral side was 22 cm^3^ and 16 cm^3^, respectively.

**Figure 2:**
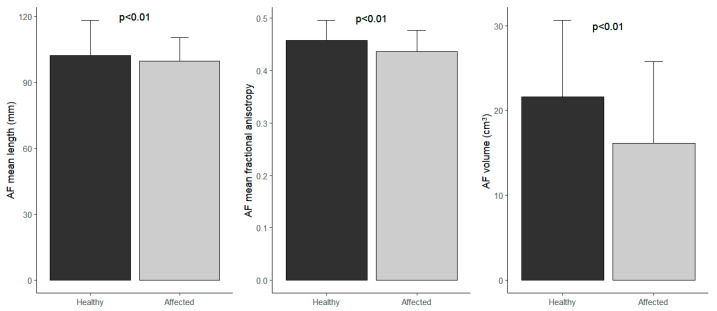
Differences between means of AF measures in the healthy and affected sides.

All these quantitative measures of the AF in the healthy and affected side are summarized in Table 1. Additionally, these differences are displayed in Figure 2.

Considering the overall sample (n = 156), Student’s *t*-test showed a significant difference between the healthy hemisphere and the hemisphere harboring tumor for AF mean length (*p*-value = 0.01), mean FA, and its volume (*p*-value < 0.001). Considering only patients with GBM (n = 98), Student’s *t*-test confirmed the results for the mean FA and volume (*p*-value < 0.001). Student’s *t*-test, evaluating LGG patients (n = 26), showed no significant difference between the healthy hemisphere and the hemisphere harboring tumor for AF mean length, mean FA, and volume (*p*-value 0.5, *p*-value 0.3, *p*-value < 0.1, respectively). In patients affected by brain metastasis (n = 18), Student’s *t*-test showed a significant difference for FA (*p*-value = 0.003). No differences were found in patients affected by meningiomas (n = 14).

- Regression analysis

Hemisphere status, healthy vs. affected side, was significantly associated with the AF mean length; in particular, the healthy side had a significantly higher AF mean length than the hemisphere harboring tumor (adj. β = 2.5, 95% CI 0.62, 4.5, *p* = 0.01). Tumor histology was significantly associated with AF mean length; in particular, LGG had a significantly lower AF mean length than GBM (adj. β =−9.3, 95% CI −15, −3.1, *p* = 0.003), while meningioma had an AF mean length significantly higher (adj. β = 19, 95% CI 14, 25, *p* < 0.001). EN volume was also significantly associated with AF mean length (adj. β = −0.19, 95% CI −0.29, −0.08, *p* < 0.001).

Hemisphere status was also significantly associated with AF mean FA; the healthy side had a significantly higher AF volume than the contralateral side (adj. β = 0.2, 95% CI 0.01, 0.03, *p* ≤ 0.001).

Patient’s gender was significantly associated with AF mean FA; males had a significantly higher AF mean FA than females (adj. β = 0.01, 95% CI 0.00, 0.08, *p* ≤ 0.013).

Hemisphere status was also significantly associated with AF volume; the healthy side had a significantly higher AF volume than the contralateral side (adj. β = 5.4, 95% CI 3.9, 7, *p* < 0.001).

Patient’s gender was significantly associated with AF volume; males had a significantly higher AF volume than females (adj. β = 3.1, 95% CI 0.67, 5.5, *p* = 0.013).

Tumor histology was significantly associated with AF volume; LGG had a significantly higher AF volume than GBM (adj. β = 5.2, 95% CI 0.56, 9.8, *p* = 0.028), while meningioma had a significantly lower AF volume (adj. β = −5.9, 95% CI −10, −1.6, *p* = 0.008).

FLAIR volume was significantly associated with AF volume (adj. β = −0.05, 95% CI −0.09, −0.01, *p* < 0.026) (Table 2).

The multiple mixed-effect linear regression conducted on the subgroup of GBM showed that the healthy side had a significantly higher AF mean FA and volume than the contralateral side (adj. β = 0.02, 95% CI 0.02, 0.03, *p* < 0.001 and adj. β = 6.6, 95% CI 4.6, 8.6, *p* < 0.001, respectively). In these subgroups, it was shown that FLAIR volume was significantly associated with AF mean FA (adj. β = 0.0001, 95% CI 0.0000, 0.0001, *p* = 0.012) (Table 3).

### 3.1. Results

Among the 1294 patients screened, 156 fulfilled the inclusion criteria: 98 (63%) with GBM, 26 (17%) with LGG, 14 (9%) with meningiomas, and 18 (12%) with metastases. The cohort included 83 males (53%) and 73 females (47%), with a mean age of 59 years. Tumors were located in the left hemisphere in 70% and in the right hemisphere in 30% of cases. Table 1 summarizes the demographic characteristics.

The mean enhancing nodule (EN) volume was 25 cm^3^, and the mean FLAIR hyperintensity volume was 49 cm^3^.

Across the cohort, the mean AF length was 102 mm on the healthy hemisphere and 100 mm on the affected side. The mean FA was 0.46 (healthy) versus 0.44 (affected), and the mean tract volumes were 22 cm^3^ (healthy) versus 16 cm^3^ (affected). Table 1 provides all quantitative metrics.

Significant reductions were observed in AF length (*p* = 0.01), FA (*p* < 0.001), and volume (*p* < 0.001) in the pathological hemisphere. Subgroup analyses revealed significant FA and volume reductions in GBM patients (*p* < 0.001), while in LGG and meningioma cases, no significant differences were detected. Among metastases, only FA showed a significant reduction (*p* = 0.003).

### 3.2. Results of the Regression Analysis

Regression analysis confirmed that healthy hemisphere status was associated with greater AF length (adj. β = 2.5 mm, 95% CI: 0.62–4.5, *p* = 0.01), FA (adj. β = 0.02, 95% CI: 0.01–0.03, *p* < 0.001), and volume (adj. β = 5.4 cm^3^, 95% CI: 3.9–7.0, *p* < 0.001).

Histological type significantly influenced AF parameters. Compared to GBM

LGG was associated with reduced AF length (adj. β = −9.3 mm, *p* = 0.003) and larger volume (adj. β = 5.2 cm^3^, *p* = 0.028);

Meningiomas exhibited increased AF length (ad. β = +19 mm, *p* < 0.001) and reduced volume (adj. β = −5.9 cm^3^, *p* = 0.008).

Higher EN volumes were associated with shorter AF length (adj. β = −0.19 mm per cm^3^, *p* < 0.001). Additionally, larger FLAIR volumes were negatively correlated with AF volume (adj. β = −0.05 cm^3^ per cm^3^, *p* = 0.026).

In the GBM subgroup, healthy hemispheres consistently showed higher FA (adj. β = 0.02, *p* < 0.001) and greater AF volume (adj. β = 6.6 cm^3^, *p* < 0.001). FLAIR volume remained significantly associated with FA values (adj. β = 0.0001, *p* = 0.012).

## 4. Discussion

**Figure 3:**
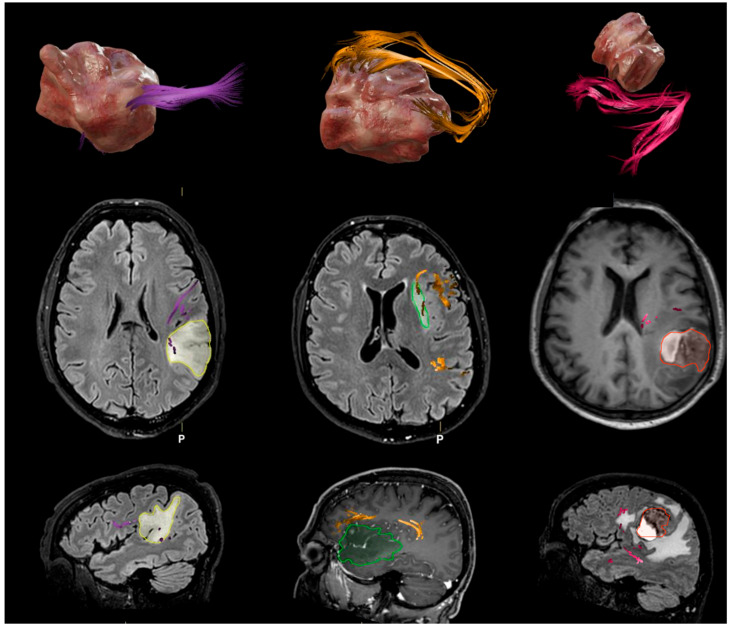
In the left column (from above to below), an LGG primarily exhibiting an infiltrative effect on the AF is visible (LGG had a significantly lower AF mean length than GBM). The middle column illustrates the mixed effect of a GBM on the AF, characterized by predominant displacement and partial infiltration. The right column shows the AF displacement due to compression by a metastasis.

This study highlighted significant morpho-volumetric alterations of the AF in patients with brain tumors located near its course. GBM is particularly highly aggressive and infiltrative and profoundly impacts overall brain structure and function, including vital white matter tracts. This destructive process leads to a significant reduction in the mean length and volume of these critical neural pathways. Given this pervasive impact, numerous attempts have been made to identify and characterize tumoral invasion in GBM by quantifying structural abnormalities within the brain’s connectome [20,21]. The collected data demonstrate a significant reduction in the length, FA, and volume of the AF in the pathological region compared to the healthy hemisphere, with marked differences in patients affected by GBM and brain metastases (Figure 3). The AF exhibits significant hemispheric asymmetry, especially concerning its morpho-volumetric characteristics and functional specialization, also in healthy subjects (for Broca’s area and Wernicke’s area connection). For instance, the horizontal curvature ranges of the AF are quite similar between the right and left hemispheres, whereas the vertical curvature ranges show that the right hemisphere generally exhibits larger ranges compared to the left hemisphere [22].

A comparison with the existing literature confirms that WM impairment in neuro-oncological patients is well-documented; however, our study provides a detailed quantification of AF alterations, particularly in terms of its length, FA, and volume [23,24,25,26,27,28,29,30]. The data obtained indicate that GBM, compared to other tumor histotypes, is associated with a more pronounced reduction in FA and AF volume, suggesting a stronger destructive effect on the integrity of WM fibers. Conversely, patients with meningioma did not show significant alterations in the analyzed parameters, indicating a lesser impact on the underlying structures compared to more invasive tumors.

Another relevant aspect emerging from this study is the relationship between AF volume and the volume of the FLAIR-positive region. The significant association between lesion volume and FA and AF volume reduction suggests a mechanism of compression and infiltration contributing to WM pathway impairment. This finding aligns with previous studies demonstrating the ability of brain tumors to alter neural connectivity through direct (infiltration) and indirect (compression and fiber displacement) effects [31,32].

Additionally, our findings resonate with our previous research on the IFOF, another major associative multitasking WM bundle involved in language and cognitive functions [33,34,35]. Like the AF, the IFOF exhibits morpho-volumetric changes due to tumor presence, with different tumor histotypes exerting varying effects. GBM, characterized by its infiltrative and destructive nature, profoundly impacts the IFOF by reducing its mean length and volume. In contrast, LGGs mainly influence the IFOF by displacing and infiltrating fibers, reflecting their slow-growing yet pervasive characteristics. Metastases, on the other hand, lead to displacement of the IFOF due to peritumoral edema rather than direct infiltration, while meningiomas, as extra-axial tumors, generally preserve the integrity of WM tracts despite inducing mechanical displacement [36].

From a clinical perspective, these alterations can significantly impact patients’ language function. The lateralization of the AF in our cohort showed a predominance of tumors in the left hemisphere, confirming the potential involvement of the language network. Although no specific functional data were collected in this cohort, reductions in FA and AF volume could be correlated with language deficits, as reported in previous studies. Similarly, previous investigations have highlighted the role of IFOF in language and attention networks, emphasizing the need for meticulous preoperative planning to preserve these crucial pathways during surgery.

The main limitations of this study include its retrospective nature and the variability in imaging acquisition parameters across different centers. Although data were normalized to reduce heterogeneity, differences in DTI protocols might have influenced the results. However, these limitations were mitigated by applying a standardized reconstruction workflow across all cases, using the same software platform and consistent ROI placement methodology. Furthermore, all tract reconstructions performed by neurosurgeons were independently reviewed and validated by expert neuroradiologists and neuroanatomists, ensuring anatomical accuracy and methodological consistency throughout the dataset. Additionally, the lack of postoperative follow-up prevents the assessment of possible AF and IFOF functional reorganization and language recovery in treated patients.

## 5. Conclusions

Our retrospective study provides new evidence on the impact of brain tumors on AF morphology, particularly in patients with GBM and metastases. Furthermore, the findings align with previous studies on IFOF alterations in the presence of different tumor types, reinforcing the importance of WM preservation in neurosurgical planning. Understanding these alterations can improve neurosurgical planning and functional prognosis, paving the way for more personalized therapeutic strategies, such as connectivity-guided surgery or targeted neurocognitive rehabilitation. Future studies, including longitudinal data and more in-depth functional evaluations, will be essential to better understand the role of the AF and IFOF in postsurgical neuroplasticity and language function preservation.

## Figures and Tables

**Table 1:** Demographic and neuroradiological data. Data reported as mean (SD) and as frequency (%).

Variable	N = 156
**Sex**	
Female	73 (47%)
Male	83 (53%)
**Age**	59 (13)
**Diagnosis**	
GBM	98 (63%)
LGG	26 (17%)
Meningioma	14 (9.0%)
Met	18 (12%)
**Location**	
Bilateral frontal	1 (0.6%)
Clinoid	1 (0.6%)
Frontal	45 (29%)
Frontal insular	2 (1.3%)
Fronto-parietal	7 (4.5%)
Fronto-temporal	4 (2.6%)
Fronto-temporo-insular	6 (3.8%)
Insular	1 (0.6%)
Occipital	1 (0.6%)
Parasagittal	1 (0.6%)
Parietal	16 (10%)
Parieto-occipital	8 (5.1%)
Parieto-temporal	4 (2.6%)
Temporal	44 (28%)
Temporal–parietal	9 (5.8%)
Temporo-insular	4 (2.6%)
Temporo-occipitale	1 (0.6%)
Thalamic	1 (0.6%)
**Side**	
Left	109 (70%)
Right	47 (30%)
**Affected arcuate fascicle mean length mm**	100 (11)
**Affected arcuate fascicle mean fractional anisotropy**	0.44 (0.04)
**Affected arcuate fascicle volume cm^3^**	16 (10)
**Healthy arcuate fascicle mean length mm**	102 (16)
**Healthy arcuate fascicle mean fractional anisotropy**	0.46 (0.04)
**Healthy arcuate fascicle volume cm^3^**	22 (9)
**Tumor volume Enhancing Nodule**	25 (21)
**Volume of FLAIR hyperintensity beyond EN**	49 (34)

**Table 2:** Analysis of the determinants of the paired mean differences between the healthy and affected brain hemispheric sides.

	Outcome: Arcuate Fascicle Mean Length (mm)			Outcome: Arcuate Fascicle Mean Fractional Anisotropy			Outcome: Arcuate Fascicle Volume cm^3^		
Characteristic	Adjusted Beta	95% CI	*p*-Value	Adjusted Beta	95% CI	*p*-Value	Adjusted Beta	95% CI	*p*-Value
Group									
Affected	—	—		—	—		—	—	
Healthy	2.5	0.62, 4.5	**0.010**	0.02	0.01, 0.03	**<0.001**	5.4	3.9, 7.0	**<0.001**
Sex									
Female	—	—		—	—		—	—	
Male	2.7	−0.51, 5.9	0.099	0.01	0.00, 0.02	**0.013**	3.1	0.67, 5.5	**0.013**
Age	−0.14	−0.30, 0.01	0.068	0.00	0.00, 0.00	0.059	0.00	−0.12, 0.12	0.994
Diagnosis									
GBM	—	—		—	—		—	—	
LGG	−9.3	−15, −3.1	**0.003**	0.01	−0.01, 0.03	0.333	5.2	0.56, 9.8	**0.028**
Meningioma	19	14, 25	**<0.001**	0.01	−0.01, 0.03	0.166	−5.9	−10, −1.6	**0.008**
Met	4.0	−1.2, 9.1	0.129	0.01	−0.01, 0.03	0.273	−2.1	−6.0, 1.7	0.278
Side									
Left	—	—		—	—		—	—	
Right	0.14	−3.4, 3.7	0.936	0.00	−0.01, 0.01	0.958	−0.29	−2.9, 2.4	0.827
Tumor Volume Enhancing Nodule	−0.19	−0.29, −0.08	**<0.001**	0.00	0.00, 0.00	0.323	0.07	−0.01, 0.14	0.097
Volume of FLAIR Hyperintensity Beyond EN	0.02	−0.03, 0.08	0.434	0.00	0.00, 0.00	0.107	−0.05	−0.09, −0.01	**0.026**

Bold was used to indicate *p*-values < 0.05.

**Table 3:** Analysis of the determinants of the paired mean differences between the healthy and affected brain hemispheric sides in the GBM subgroup.

	Outcome: Arcuate Fascicle Mean Fractional Anisotropy			Outcome: Arcuate Fascicle Volume (cm^3^)		
Characteristic	Adjusted Beta	95% CI	*p*-Value	Adjusted Beta	95% CI	*p*-Value
Group						
Affected	—	—		—	—	
Healthy	0.02	0.02, 0.03	**<0.001**	6.6	4.6, 8.6	**<0.001**
Sex						
Female	—	—		—	—	
Male	0.00	−0.01, 0.02	0.382	2.7	−0.46, 5.8	0.094
Age	0.00	0.00, 0.00	0.299	0.00	−0.16, 0.16	0.985
Side						
Left	—	—		—	—	
Right	0.00	−0.01, 0.01	0.807	−0.03	−3.5, 3.4	0.985
Tumor Volume Enhancing Nodule	0.00	0.00, 0.00	0.743	0.08	−0.02, 0.17	0.100
Volume of FLAIR Hyperintensity Beyond EN	0.0001	0.0000, 0.0001	**0.012**	−0.04	−0.09, 0.02	0.168

Bold was used to indicate *p*-values < 0.05.

## Data Availability

Data of the current original research are available from the corresponding author on reasonable request. The data have been delivered to the authors and belong to them.
